# Current perspectives of the Japanese Esophageal Oncology Group on the development of immunotherapy for esophageal cancer

**DOI:** 10.1093/jjco/hyac138

**Published:** 2022-09-01

**Authors:** Toru Kadono, Shun Yamamoto, Ken Kato

**Affiliations:** Department of Head and Neck, Esophageal Medical Oncology, National Cancer Center Hospital, Tokyo, Japan; Department of Medicine, Juntendo University Graduate School of Medicine, Tokyo, Japan; Department of Head and Neck, Esophageal Medical Oncology, National Cancer Center Hospital, Tokyo, Japan; Department of Head and Neck, Esophageal Medical Oncology, National Cancer Center Hospital, Tokyo, Japan

**Keywords:** esophageal squamous cell carcinoma, immune checkpoint inhibitor, nivolumab, pembrolizumab, pre-operative treatment, post-operative treatment

## Abstract

Esophageal cancer is the seventh most common cancer worldwide and continues to have a poor prognosis. Starting with the development of immune checkpoint inhibitors for patients with metastatic melanoma, many clinical trials have been conducted to evaluate the efficacy and safety of immune checkpoint inhibitors against various malignancies. Although few effective drugs are available for patients with advanced esophageal cancer, two immune checkpoint inhibitors, nivolumab and pembrolizumab, have been approved as second-line treatments for advanced esophageal squamous cell carcinoma. Recently, immune checkpoint inhibitors have shown promising results as post-operative therapies and first-line treatments for advanced esophageal cancer. Nivolumab has been approved as a post-operative therapy based on the CheckMate-577 trial, and nivolumab, ipilimumab and pembrolizumab have been approved as first-line treatments based on the CheckMate-648 trial and the KEYNOTE-590 trial. In addition, many trials of immune checkpoint inhibitors plus pre-operative treatment or definitive chemoradiotherapy are ongoing. The Japan Esophageal Oncology Group was established in 1978 and has conducted numerous clinical trials, most of which have examined multimodality treatments. In the era of immunotherapy, Japan Esophageal Oncology Group is conducting a clinical trial studying multimodality treatment with an immune checkpoint inhibitor. JCOG1804E (FRONTiER) is a phase I trial to evaluate the safety and efficacy of nivolumab plus pre-operative chemotherapy followed by surgery. These results might improve the clinical outcomes of esophageal cancer patients.

## Teaser

Nivolumab and pembrolizumab have become the standard of care for the second-line treatment of advanced esophageal squamous cell carcinoma. Multiple clinical trials assessing immune checkpoint inhibitor-containing treatments for not only advanced cases but also for locally advanced cases are ongoing. One such example is the JCOG1804E trial.

## Introduction

In 2018, the estimated number of new esophageal cancer (EC) cases worldwide was 572 000, and 509 000 deaths as a result of EC were thought to have occurred. Among cancer types, EC ranks seventh in terms of incidence and sixth in terms of mortality (1). EC is mainly divided into two major histological subtypes: squamous cell carcinoma (SCC) and adenocarcinoma (AC).

Worldwide, patients with esophageal SCC (ESCC) account for about 87% of all EC patients. However, the dominant histological subtypes differ according to geographical region and culture. For example, in North America and Europe, the most common histological subtype is esophageal AC (EAC); in Eastern Asian countries, the most common histological subtype is ESCC (1). In Japan, ESCC patients account for 90% of all EC patients (2,3); consequently, the Japan Esophageal Oncology Group (JEOG) has focused on the development of treatments (mainly multi-modal) for ESCC. Treatment drugs for EC are limited, and the prognosis in patients with EC remains poor. Recently, immune checkpoint inhibitors (ICIs) have been developed in various cancers and shown antitumor activity in EC. Here, we show the development of ICIs in EC according to disease stage and discuss the prospect of ICIs Table 1 and 2.

**Table 1:** Results of phase III clinical trials evaluating ICIs for EC

Agent (Trial)	Line	Location	Histology	No. of pts	Regimen	Response rate	Median PFS	Median OS	Ref
**Metastatic or recurrent setting**									
Nivolumab (ATTRACTION-3)	2	E	SCC	419	Nivolumab	19%	1.7 months	10.9 months	(41)
					PTX or DTX	22%	3.4 months	8.4 months	
Pembrolizumab (KEYNOTE-181)	2	E/EGJ	SCC/AC (SCC 63%)	628	(All patients)				(49)
					Pembrolizumab	13.1%	2.1 months	7.1 months	
					PTX or DTX or CPT-11	6.7%	3.4 months	7.1 months	
					(CPS ≥10)				
					Pembrolizumab	21.5%	2.6 months	9.3 months	
					PTX or DTX or CPT-11	6.1%	3.0 months	6.7 months	
					(SCC)				
					Pembrolizumab	16.7%	2.2 months	8.2 months	
					PTX or DTX or CPT-11	7.4%	3.1 months	7.1 months	
Camrelizumab (ESCORT)	2	E	SCC	457	Camrelizumab	20.2%	1.9 months	8.3 months	(57)
					DTX or CPT-11	6.4%	1.9 months	6.2 months	
Tislelizumab (RATIONALE-302)	2	E	SCC	512	Tislelizumab	20.3%	-	8.6 months	(58)
					PTX or DTX or CPT-11	9.8%	-	6.3 months	
Pembrolizumab (KEYNOTE-590)	1	E/EGJ	SCC/AC (SCC 73%)	749	(All patients)				(50)
					CF + Pembrolizumab	45.0%	6.3 months	12.4 months	
					CF	29.3%	5.8 months	9.8 months	
					(CPS ≥10)				
					CF + Pembrolizumab	51.1%	7.5 months	13.5 months	
					CF	26.9%	5.5 months	9.4 months	
					(ESCC)				
					CF + Pembrolizumab	43.8%	6.3 months	12.6 months	
					CF	31.0%	5.8 months	9.8 months	
Nivolumab (CheckMate-648)	1	E	SCC	970	(PD-L1 ≥ 1%)				(56)
					CF + Nivolumab	53%	6.9 months	15.4 months	
					Nivolumab + Ipilimumab	35%	4.0 months	13.7 months	
					CF	20%	4.4 months	9.1 months	
**Resectable, locally advanced setting**									
Nivolumab (CheckMate-577)	Adjuvant	E/EGJ	SCC/AC (SCC 29%)	794	Nivolumab	-	22.4 months^a^	NE	(46)
					Placebo	-	11.0 months^a^	NE	

Abbreviations: pts, patients; E, esophagus; EGJ, esophagogastric junction; SCC, squamous cell carcinoma; AC, adenocarcinoma; CF, cisplatin plus fluorouracil; CPS, combined positive score; ESCC, esophageal squamous cell carcinoma; NE, not evaluated.

^a^Disease-free survival

## ICIs for advanced EC

Systemic chemotherapy is required for patients with recurrences or metastasis to palliate symptoms and improve survival. Although few studies have validated the efficacy of palliative chemotherapy for EC, chemotherapy combined with platinum and fluoropyrimidine was recognized as a standard therapy (3,4,20,21).

The recent development of ICIs, such as anti-cytotoxic T-lymphocyte antigen 4 (CTLA-4) and anti-programmed death-1 (PD-1), was a breakthrough in cancer treatment. Tumor-specific antigen is expressed on tumor cells as a result of genetic and epigenetic alterations; these antigens are recognized by dendritic cells or antigen-presenting cells. Subsequently, the antigens are presented to T-cells, and activated T-cells kill the tumor cells. In an interaction between T-cells and tumor cells, the degree of the T-cell response is regulated by a balance between activating and inhibitory signals, known as immune checkpoints (24,25). PD-1 expressed on the surface of T-cells interacts with programmed death-ligand 1 (PD-L1) on cancer cells and immune cells, downregulating T-cell activation and leading to T-cell apoptosis (26,27). Therefore, blockade of the PD-1/PD-L1 pathway produces anti-tumor effects, and PD-1/PD-L1 inhibitors have conferred clinical benefits in patients with various cancers (28–38).

**Table 2:** Ongoing trials of ICIs for EC

Trial	Agent	Line	Phase	No. of pts	Treatment Arm(s)		Ref
**Metastatic or recurrent setting**							
RATIONALE-306	Tislelizumab	1	III	649	Chemotherapy + Tislelizumab		
					Chemotherapy		
LEAP-014	Pembrolizumab	1	III	862	Pembrolizumab + Lenvatinib + Chemotherapy		
					Pembrolizumab + Chemotherapy		
**Unresectable, locally advanced setting**							
TENERGY	Atezolizumab	Following dCRT	II	50	dCRT followed by Atezolizumab		(60)
NOBEL	Nivolumab	Combined with dCRT	II	60	dCRT + Nivolumab		
SKYSCRAPER-07	Tiragolumab Atezolizumab	Following dCRT	III	750	dCRT followed by	Tiragolumab + Atezolizumab	
					Placebo + Atezolizumab		
					Double Placebo		
KUNLUN	Durvalumab	Combined with dCRT	III	600	dCRT + Durvalumab		
KEYNOTE-975	Pembrolizumab	Combined with dCRT	III	600	dCRT + Pembrolizumab		(61)
**Resectable, locally advanced setting**							
CRUCIAL	Nivolumab	Combined with dCRT	II	130	dCRT + Nivolumab		
					dCRT + Nivolumab + Ipilimumab		
FRONTiER (JCOG1804E)	Nivolumab	Neoadjuvant	I	36	CF + Nivolumab		(72)
					DCF + Nivolumab		
					FLOT + Nivolumab		

Abbreviations: pts, patients; dCRT, definitive chemoradiotherapy; CF, cisplatin plus fluorouracil; FLOT, docetaxel, oxaliplatin, leucovorin plus fluorouracil.

### Nivolumab

Nivolumab is a humanized monoclonal IgG4 PD-1 antibody. The ATTRACTION-1 phase II trial assessed the efficacy and safety of nivolumab monotherapy for patients with advanced EC refractory or intolerant to standard therapies, such as fluoropyrimidine and platinum or taxane. In 65 ESCC patients, the ORR, which was the study’s primary endpoint, was 17% (95% CI: 10–28%), and the median PFS and OS were 1.5 months (95% CI: 1.4–2.8 months) and 10.8 months (95% CI: 7.4–13.3 months), respectively. In addition, nivolumab had a manageable safety profile, with grade 3–4 adverse events reported in 26% of the patients. The most common adverse events in any grade were diarrhea (20%), decreased appetite (18%), lung infection (18%), constipation (11%), rash (11%) and fatigue (11%) (39). The 5-year follow-up data were reported in ASCO-GI 2021. The median OS and the median PFS were 10.8 months (95% CI: 7.4–13.9 months) and 1.5 months (95% CI: 1.4–2.8 months), respectively (40).

Based on the promising results of the ATTRACTION-1 trial, the ATTRACTION-3 phase III trial was performed. This study was a multicenter, randomized, open-label, phase III trial performed in Asia and Western countries. Patients who had previously received fluoropyrimidine- and platinum-based chemotherapy with ESCC were randomized to nivolumab monotherapy or taxane monotherapy. The median OS was statistically longer in the nivolumab group (10.9 months, 95% CI: 9.2–13.3 months) than in the chemotherapy group (8.4 months, 95% CI: 7.2–9.9 months) with an HR of 0.77 (95% CI: 0.62–0.96, *P* = 0.019), but the median PFS and ORR were not superior in the nivolumab group (1.7 months and 19%), compared with the chemotherapy group (3.4 months and 22%). A clinical survival benefit with nivolumab was observed regardless of tumor PD-L1 expression. PD-L1 status in this study was evaluated by tumor proportion score. Grade 3–4 treatment-related adverse events were reported in 18% of the patients in the nivolumab group, compared with 63% of the patients in the chemotherapy group, and the most common adverse events were comparable to those seen in the ATTRACTION-1 trial (41). In ASCO-GI 2021, the 3-year follow-up of the ATTRACTION-3 trial was reported. Nivolumab showed a continued efficacy with a 2-year OS of 20.2% and a 3-year OS of 15.3%, compared with 13.5 and 8.7% in the chemotherapy group, respectively (42). Based on these results for the ATTRACTION-3 trial, nivolumab monotherapy was approved as a second-line treatment for patients with advanced ESCC.

Nivolumab monotherapy have become the second-line standard treatment, this treatment showed progressive disease at the best response of about half of ESCC patients and no useful biomarkers were detected in the ATTRACTION-3 trial. Therefore, the biomarker analysis was needed for this population. In the KEYNOTE-180 and KEYNOTE-181 trials, PD-L1 expression using combined positive score was a promising biomarker for pembrolizumab monotherapy; there were no data on PD-L1 (CPS) and efficacy of nivolumab monotherapy. Regarding this clinical question, a retrospective study showed a trend toward a better PFS with a higher CPS cut-off (CPS 5: HR, 1.33: CPS 10: HR, 0.85; CPS 20: HR, 0.70). Therefore, CPS might be a potential biomarker for evaluating the efficacy of nivolumab in patients with advanced ESCC (44). Additionally, further studies are needed to identify optimal biomarkers other than CPS for nivolumab, and JEOG members are presently conducting the ANTARES study (UMIN000043703) to explore useful new biomarkers using blood, biopsy and fecal samples.

Following the success of nivolumab monotherapy for patients with advanced EC, nivolumab combined with chemotherapy or ipilimumab which is an anti-CTLA-4 monoclonal antibody was developed. CTLA-4 is expressed on T-cells and regulates T-cell activation by counteracting and inhibiting CD28. Interactions between CTLA-4 and CD28 inactivate T-cells (54). The CheckMate-648 phase III trial examined nivolumab plus ipilimumab or nivolumab plus CF versus CF as a first-line chemotherapy in patients with metastatic or recurrent ESCC. The primary endpoints were OS and PFS in patients with PD-L1 ≥ 1%. The nivolumab plus CF arm had a significantly longer OS (15.4 months vs. 9.1 months, HR: 0.54, 99.5% CI: 0.37–0.80, *P* < 0.0001) and PFS (6.9 months vs. 4.4 months, HR: 0.65, 98.5% CI: 0.46–0.92, *P* = 0.0355) than the CF arm in patients with PD-L1 ≥ 1%. The nivolumab plus ipilimumab arm also had a significantly longer OS (13.7 months vs. 9.1 months, HR: 0.64, 98.6% CI: 0.46–0.90, *P* = 0.001) than the CF arm in patients with PD-L1 ≥ 1%, but the PFS was comparable (4.0 months vs. 4.4 months, HR: 1.02, 98.5% CI: 0.73–1.43, *P* = 0.8958) (56). The combination of nivolumab plus chemotherapy and nivolumab plus ipilimumab were approved as a first-line treatment for patients with advanced EC regardless of PD-L1 expression in May 2022.

### Pembrolizumab

Pembrolizumab is another humanized IgG4 monoclonal PD-1 antibody that was first evaluated in the KEYNOTE-028 phase I trial of patients with PD-L1-positive advanced solid tumors. In this trial, 78% of the patients had ESCC, and 87% patients had received two or more prior lines of chemotherapy. The ORR, median PFS and OS were 30% (95% CI:13–53%), 1.8 months (95% CI: 1.7–2.9 months) and 7.0 months (95% CI: 4.3–17.7 months), respectively (47).

The KEYNOTE-180 phase II trial to evaluate the efficacy and safety of pembrolizumab was conducted for patients who received two or more prior lines of chemotherapy with advanced metastatic ESCC or EAC. The proportion of ESCC was 52.1%, and 47.9% of patients had PD-L1 positive tumors (PD-L1-positivity was defined as CPS ≥10). The ORR, median PFS and OS were 9.9% (95% CI: 5.2–16.7%), 2.0 months (95% CI: 1.9–2.1 months) and 5.8 months (95% CI: 4.5–7.2 months), respectively (48). Grade 3–5 treatment-related adverse events were reported in 12.4% of the patients, and the most common adverse events in any grade were fatigue, rash, pruritus, hypothyroidism and diarrhea. Since pembrolizumab showed a promising response and manageable safety profile in the phase II trial, the KEYNOTE-181 phase III trial was conducted. Patients who had one prior line of standard chemotherapy with ESCC or EAC were randomized to receive pembrolizumab or the investigator’s choice of chemotherapy (paclitaxel, docetaxel or irinotecan) as a second-line in this randomized, open-label, global phase III trial. The primary endpoints were OS in patients with CPS ≥10, in patients with ESCC and in all the patients. In this trial, the proportions of Asians, patients with ESCC and patients with a CPS ≥10 were 38.6, 63.8 and 35.3 at baseline, respectively. The pembrolizumab arm in patients with a CPS ≥10 had a better median OS, compared with the chemotherapy arm (9.3 months vs. 6.7 months, HR: 0.69, 95% CI: 0.52–0.93, *P* = 0.0074); however, the median OS of the ESCC patients was not superior to that for the chemotherapy arm (8.2 months vs. 7.1 months, HR: 0.78, 95% CI: 0.63–0.96, *P* = 0.0095). Grade 3–5 treatment-related adverse events were reported in 18% of patients in the pembrolizumab arm, compared with 40.9% of the patients in the chemotherapy arm (49). Based on these results, pembrolizumab was approved as a second-line treatment for recurrent, locally advanced or metastatic ESCC with a CPS ≥10.

These results suggested that chemotherapy combined with pembrolizumab as a first-line chemotherapy might improve patient outcome. The KEYNOTE-590 phase III trial was a randomized, double-blind, placebo-controlled trial evaluating chemotherapy with CF plus pembrolizumab versus chemotherapy for patients with locally advanced unresectable or metastatic EC or esophagogastric junction AC. The primary endpoints were OS in patients with ESCC and a CPS ≥10, OS and PFS in patients with ESCC, OS and PFS in patients with a CPS ≥10, and OS and PFS in all the patients. Seven hundred and forty-nine patients were assigned, and the proportion of patients with SCC was 73.5% in the chemotherapy plus pembrolizumab arm and 72.9% in the chemotherapy arm. The chemotherapy plus pembrolizumab arm had a better median OS than the chemotherapy arm in all the patients (12.4 months vs. 9.8 months, HR: 0.73, 95% CI: 0.62–0.86, *P* < 0.0001), in the CPS ≥10 cohort (13.5 months vs. 9.4 months, HR: 0.62, 95% CI: 0.49–0.78, *P* < 0.0001), in the patients with ESCC (12.6 months vs. 9.8 months, HR: 0.72, 95% CI: 0.60–0.88, *P* < 0.0006) and in the patients with ESCC and a CPS ≥10 (13.9 months vs. 8.8 months, HR: 0.57, 95% CI: 0.43–0.75, *P* < 0.0001). The median PFS was also better in the chemotherapy plus pembrolizumab arm in all the patients (6.3 months vs. 5.8 months, HR: 0.65, 95% CI: 0.55–0.76, *P* < 0.0001), in the CPS ≥10 cohort (7.5 months vs. 5.5 months, HR: 0.51, 95% CI: 0.41–0.65, *P* < 0.0001) and in the ESCC patients (6.3 months vs. 5.8 months, HR: 0.65, 95% CI: 0.54–0.78, *P* < 0.0001). Grade 3–5 treatment-related adverse events were reported in 72% of the patients in the chemotherapy plus pembrolizumab arm, compared with 68% of patients in the chemotherapy arm (50). The combination of chemotherapy plus pembrolizumab was approved as a first-line treatment for patients with advanced EC regardless of CPS score in November 2021.

The results of the above trials for pembrolizumab and nivolumab suggest that the histological subtype might be a predictive marker of the efficacy of ICIs for patients with metastatic EC. A subgroup analysis in the KEYNOTE-181 trial showed that patients with ESCC seemed to have better survival benefits (HR: 0.77, 95% CI: 0.63–0.96) than those with EAC (HR: 1.12, 95% CI: 0.85–1.47) (49). In addition, the CheckMate-577 trial showed a similar tendency for survival benefits in ESCC (HR: 0.61, 95% CI: 0.42–0.88) and EAC (HR: 0.75, 95% CI: 0.59–0.96) (46). Some biological studies have shown the occurrence of ESCC to be associated with smoking, and smoking is strongly associated with high PD-L1 expression and a high tumor mutational burden (51–53). These results may explain why ESCC patients receiving ICIs experience a greater clinical benefit than EAC patients. However, on the other hand, the KEYNOTE-590 trial reported a similar benefit between ESCC patients (HR: 0.72, 95% CI: 0.60–0.88) and EAC patients (HR 0.74, 95% CI: 0.52–1.02). Consistent biological mechanisms according to histology have not been established, and further biological studies are warranted.

### Other ICIs and current treatment developments

Camrelizumab is an anti-PD-1 IgG4 antibody that was investigated for patients with metastatic or recurrent ESCC in China. The phase III ESCORT trial compared camrelizumab with a regimen of the investigator’s choice with docetaxel or irinotecan as the second-line treatment. A total of 457 patients were randomly allocated, and the median OS was superior in the camrelizumab arm (8.3 months, 95% CI: 6.8–9.7 months), compared with the chemotherapy arm (6.2 months, 95% CI: 5.7–6.9 months), with an HR of 0.71 (95% CI: 0.57–0.87, *P* = 0.001) (57). In addition, the RATIONALE-302 phase III trial compared tislelizumab, which is an anti-PD-1 antibody, with chemotherapy (paclitaxel, docetaxel or irinotecan) as a second-line chemotherapy for patients with metastatic or recurrent ESCC. Overall, 512 patients were randomized to each group, and the tislelizumab arm had a significantly longer OS than the chemotherapy arm in the intention-to-treat population (8.6 months vs. 6.3 months, HR: 0.70, 95% CI: 0.57–0.85, *P* = 0.0001) (58).

As ongoing trials, the RATIONALE-306 phase III is to evaluate the safety and efficacy of tislelizumab plus chemotherapy (platinum plus fluorouracil, platinum plus capecitabine or platinum plus paclitaxel) versus chemotherapy as a first-line chemotherapy for patients with metastatic or recurrent ESCC (NCT03783442). Moreover, the LEAP-014 phase III (NCT04949256) evaluating the safety and efficacy of addition of lenvatinib to pembrolizumab, fluorouracil and platinum as a first-line chemotherapy is ongoing. As a second or later line chemotherapy, a multi-cohort phase II study of regorafenib plus nivolumab for metastatic solid tumors including EC (NCT04704154) and phase Ib study of futibatinib plus pembrolizumab for FGFR-positive solid tumors (JapicCTI-195 063) are ongoing.

## ICIs for unresectable locally advanced EC

For patients with unresectable locally advanced ESCC, definitive CRT of CF plus 60 Gy is the standard treatment based on the JCOG9516 study in Japan (3,4,17).

Novel treatment strategies consisting of definitive CRT followed by an ICI for unresectable locally advanced EC are being investigated based on the results of the phase III PACIFIC trial comparing CRT followed by durvalumab, which is an anti-PD-L1 antibody, with CRT alone for patients with unresectable locally advanced NSCLC, since this trial demonstrated a superior PFS (HR: 0.51, 95% CI: 0.41–0.63) and OS (HR: 0.68, 99.73% CI: 0.47–0.997, *P* = 0.0025) for the CRT plus durvalumab arm (59). For patients with unresectable locally advanced ESCC without distant metastasis, the phase II TENERGY trial to evaluate the safety and efficacy of definitive CRT followed by atezolizumab, which is an anti-PD-L1 antibody (UMIN000034373) (60), the phase II NOBEL trial to evaluate the safety and efficacy of definitive CRT with CF plus nivolumab followed by sequential nivolumab (UMIN000035889), the phase III SKYSCRAPER-07 trial comparing definitive CRT followed by tiragolumab, which is an anti-TIGIT antibody, plus atezolizumab; a tiragolumab-matched placebo plus atezolizumab; and a double placebo (NCT04543617), and the phase III KUNLUN trial comparing concurrent durvalumab and definitive CRT with a placebo (NCT04550260) are ongoing. In addition, for patients with unresectable locally advanced EC, the phase III KEYNOTE-975 trial comparing one initial administration of pembrolizumab and concurrent pembrolizumab plus definitive CRT with a placebo plus definitive CRT is ongoing (61).

## ICIs for resectable locally advanced EC

### Standard treatment for resectable locally advanced EC in Japan

For patients with clinical Stage I without lymph node metastasis (cT1N0M0) (Union for International Cancer Control [UICC] 8th edition), endoscopic resection can be performed if the tumor depth is limited to the mucosal layer. An esophagectomy is the standard treatment if the tumor depth exceeds the mucosal layer or if the size of the tumor is greater than three-fourths of the luminal circumference (3,4). For patients who refuse surgery or are intolerant to surgery, definitive chemoradiotherapy (CRT) seems to be a treatment option from the result of JCOG0502 study (7).

For patients with resectable locally advanced EC (clinical Stage I [cT1N1M0], II, III, IVA [cT1-3N3M0]), pre-operative chemotherapy with CF was the standard treatment based on the results of the JCOG9907 trial, which compared this treatment against post-operative CF chemotherapy (3,4). Recently, the JCOG1109 phase III study comparing both pre-operative DCF and pre-operative CRT with pre-operative CF for the treatment of locally advanced ESCC has been conducted. This study showed a superior OS for the DCF arm, compared with the CF arm, but did not show a superior OS for the CF-RT arm, compared with the CF arm. Based on this study, DCF therapy followed by surgery for resectable ESCC patients is standard treatment in Japan (12). Definitive CRT was as an option for patients who refuse surgery or is unsuitable for surgery because of complications from the result of JCOG0909 study (3,4,15).

The standard treatments for advanced or unresectable locally advanced ESCC are almost the same in Japan and Western countries. However, the standard treatment for resectable locally advanced ESCC differs. The standard treatment in Western countries consists of pre-operative CRT plus an esophagectomy, based on the results of the CROSS trial (10).

### Nivolumab

The high recurrence rate of resectable locally advanced EC, even after pre-operative CRT, remains a problem, especially for EC without pathological complete resection (45). Nivolumab has been developed as a post-operative therapy to improve the outcome of patients with resectable locally advanced EC. The phase III, global, randomized, double-blind, placebo-controlled CheckMate 577 trial compared nivolumab with a placebo as a post-operative treatment. Patients with stage II/III EC or esophagogastric junction cancer with AC or SCC receiving pre-operative CRT followed by complete resection and with confirmed residual pathological disease were randomized to receive nivolumab or the placebo. The primary endpoint was the disease-free survival (DFS). A total of 794 patients were assigned, and patients with SCC accounted for 29% of the patients in each group. The nivolumab group had a significantly better median DFS compared with the placebo group (22.4 months vs. 11.0 months, HR: 0.69, 96.4% CI: 0.56–0.86, *P* < 0.001). The DFS was longer in the nivolumab group regardless of the histological type. Grade ≥ 3 treatment-related adverse events were 13% in the nivolumab group and 6% in the placebo group. The most common adverse events were fatigue, diarrhea, pruritus and rash in the nivolumab group (46).

Post-operative nivolumab monotherapy conferred clinical benefits in the CheckMate 577 trial; however, its use as a standard treatment in Japan remains problematic. The standard treatment for resectable locally advanced ESCC in Japan is pre-operative chemotherapy with DCF and esophagectomy with D2-3 lymph node dissection based on the JCOG1109 trial (12). On the other hand, in the CheckMate 577 trial, CRT was used as a pre-operative therapy, and the main histology was AC; as well, the types of surgery included not only esophagectomy but also proximal, total or distal gastrectomy with D0-3 lymph node dissection. Because of these differences, novel evidence supporting post-operative nivolumab monotherapy in Japan is still needed. Additionally, no data on OS has been reported. Therefore, further data about the survival benefits or other ongoing clinical trials related to perioperative treatments are needed.

### JCOG1804E (FRONTiER) trial

The clinical benefits of nivolumab for patients with ESCC were demonstrated in the ATTRACTION-3 trial, and some trials for non-small cell lung cancer (NSCLC) have shown the efficacy of nivolumab monotherapy and a combination of nivolumab and cytotoxic chemotherapy as a pre-operative therapy (68,69). In addition, the use of an ICI pre-operatively showed a greater efficacy than when used post-operatively in a pre-clinical study (70).

In Japan, the standard pre-operative treatment for resectable, locally advanced ESCC was pre-operative CF followed by surgery; however, pre-operative CF therapy resulted in a histopathological complete response (pCR) rate of only 5% (8). Pre-operative DCF therapy resulted in a superior OS, compared with pre-operative CF therapy, in the JCOG1109 trial. Additionally, not for ESCC patients but for EAC or gastric cancer patients, peri-operative fluorouracil and leucovorin, oxaliplatin and docetaxel (FLOT) therapy has been established as a standard treatment in Western countries (71).

Given this background, not only doublet chemotherapy plus nivolumab but also triplet chemotherapy plus nivolumab are promising treatments. To improve the clinical outcomes of patients with resectable locally advanced ESCC, a multi-cohort phase I study, the JCOG1804E (FRONTiER) study, evaluating the safety and efficacy of pre-operative treatment with nivolumab plus CF, DCF or FLOT is ongoing (72).

First, 24 patients will be divided into cohorts of 6 people each (cohorts A–D) to evaluate safety. Cohort A will receive two courses of cisplatin (80 mg/m^2^) and nivolumab (360 mg/body) on day 1 and fluorouracil (800 mg/m^2^) on days 1–5 every 3 weeks. Cohort B will receive one prior administration of nivolumab (240 mg/body) 2 weeks before the start of chemotherapy followed by the same regimen as that used in cohort A. Cohort C will receive three courses of docetaxel (70 mg/m^2^), cisplatin (70 mg/m^2^) and nivolumab (360 mg/body) on day 1 and fluorouracil (750 mg/m^2^) on days 1–5 every 3 weeks. Cohort D will receive one prior administration of nivolumab (240 mg/body) 2 weeks before the start of chemotherapy followed by the same regimen as that used in cohort C. Subsequently, an esophagectomy with 2–3 field lymph node dissection will be performed within 84 days of the last dose of pre-operative chemotherapy. Next, 12 patients will be added to cohort E, which will receive four courses of docetaxel (50 mg/m^2^), oxaliplatin (85 mg/m^2^), leucovorin (200 mg/m^2^), fluorouracil (2600 mg/m^2^) and nivolumab (240 mg/body) on day 1 every 2 weeks. The primary endpoint will be the incidence of dose-limiting toxicities (DLTs) from the initial dose until the 30th post-operative day, and the secondary endpoints will be the ORR during pre-operative chemotherapy, the pCR rate, the proportion of curative resections, the rate of protocol treatment completion, the PFS/OS and the frequency of adverse events.

The short-term results for cohorts A and B were reported at ASCO-GI 2021. In total, 13 patients were registered in cohort A (*n* = 6) and cohort B (*n* = 7). In these cohorts, no DLTs were observed in 12 patients, and one patient in cohort B was excluded because a non-residual resection was not obtained. Grade 3 or more adverse events consisted of neutropenia during pre-operative treatment (46.3%) and anastomotic leakage (8.3%) after surgery. One patient in cohort B had grade 2 adrenal insufficiency; no treatment-related deaths were reported. The R0 resection rate was 92.3% (12/13), and the pathological complete response rate was 33.3% (2/6) in cohort A (73).

Additionally, the short-term results for cohorts C and D were reported at ASCO-GI 2022. In total, 12 patients were registered in cohort C (*n* = 6) and cohort D (*n* = 6). No DLTs were observed in cohort C, but one patient in cohort D developed a grade 3 rash and dyspnea. The R0 resection rate was 91.7% (11/12), and the pathological complete response rate was 33.3% (4/12) in both cohorts (74).

Based on the results of cohorts A–D, pre-operative CF or DCF plus nivolumab therapy followed by surgery appears to be well tolerated and to show promising efficacy. However, detailed data on biomarkers or cohort E (pre-operative FLOT plus nivolumab) have not yet been reported, and further investigations are expected.

### Current treatment development

A phase I trial to assess the safety, feasibility and efficacy of induction nivolumab prior to CRT plus nivolumab as a neoadjuvant therapy followed by an esophagectomy in patients with stage II/III EC or esophagogastric cancer was conducted. In total, 12 out of 16 patients (75.0%) had adverse events related to any treatment, and 4 patients (25.0%) had grade 3 adverse events of dyspnea, upper respiratory tract infection, transaminitis and rash. The pCR rates for EAC and ESCC were 28.6% (4/14) and 50.0% (1/2), respectively (62). A phase II trial to assess the safety, feasibility and efficacy of neoadjuvant CRT plus pembrolizumab and adjuvant pembrolizumab following surgery for patients with resectable advanced ESCC was also conducted. Although the pCR rate was 46.1%, one deaths occurred before surgery (hematemesis) and two deaths occurred after surgery (acute lung injury) (63). The phase II PERFECT trial examined pre-operative CRT with atezolizumab followed by surgery for patients with resectable EAC. The primary endpoint was the ratio of patients who completed a treatment that included atezolizumab. Thirty-nine patients were enrolled, and 24 patients completed the pre-operative treatment; the pCR rate (Mandard 1) was 39% (9/23) (64). A phase I/II trial to assess the safety and efficacy of pre-operative CRT with avelumab, which is an anti-PD-L1 antibody, followed by surgery plus post-operative avelumab in patients with clinical stage cT1N1M0, cT2-3 N0-2 M0 had a pCR rate of 43% (3/7) and one patient developed grade 2 hypothyroidism (65).

For patients with locally advanced EC who were not candidates for primary surgery, the phase II CRUCIAL trial to evaluate the efficacy and safety of definitive CRT with FOLFOX plus nivolumab followed by sequential nivolumab and CRT plus nivolumab and ipilimumab followed by sequential nivolumab and ipilimumab (NCT03437200) is ongoing.

## Conclusion

ICIs have improved the treatment outcomes and have changed the treatment strategies for EC. In Japan, chemotherapy plus nivolumab and nivolumab plus ipilimumab have been approved as first-line treatments based on the CheckMate-648 study. The superiority of pembrolizumab as a second-line treatment in the KEYNOTE-181 trial was limited to patients with a CPS ≥10; however, the KEYNOTE-590 trial showed a superior OS and PFS for the chemotherapy plus pembrolizumab group, compared with the chemotherapy group, as a first-line treatment. The chemotherapy plus pembrolizumab has been approved as a first-line treatment regardless of CPS score in Japan. Some trials of anti-PD-1/PD-L1 inhibitors for resectable/unresectable locally advanced EC, including the JCOG1804E trial, are ongoing.

## Conflict of interest statement

Dr Ken Kato has received research grants from ONO PHARMACEUTICAL, Shionogi, MSD, Merck Serono, Beigene, Oncolys Biopharma, Chugai Pharmaceutical and BAYER outside the submitted work. Toru Kadono and Shun Yamamoto have nothing to declare.
